# Habitat selection and human aesthetic responses to flowers

**DOI:** 10.1017/ehs.2020.66

**Published:** 2021-01-11

**Authors:** Martin Hůla, Jaroslav Flegr

**Affiliations:** Charles University, Faculty of Science, Prague, Czech Republic

**Keywords:** evolutionary aesthetics, habitat selection, flower preference, perception of flowers

## Abstract

Although the aesthetic appreciation of flowers is a well-known aspect of human behaviour, theories explaining its origin are missing. The only exception is the evolutionary theory of Heerwagen and Orians. Surprisingly, it has not yet been empirically tested. The authors suggest that humans aesthetically respond to flowers because they signal food availability. The logic of the theory implies that fruits are more reliable and direct food availability signals than flowers. Therefore, fruits should elicit stronger aesthetic responses than flowers. To test this assumption, we performed two online studies in the Czech Republic. The participants (*n =* 2792 and 744, respectively) indicated on a six-point scale their aesthetic response to photographs of 14 edible Czech plant species (study A) and 20 edible plant species from the African savannas (study B), varying in growth stage (flowering, fruiting). We found no difference between the Czech fruiting and flowering plants and a stronger aesthetic response to African flowering plants. A third study (*n =* 817) confirmed that flowers were preferred to fruits, using a forced-choice paradigm. Our results suggest that the theory cannot fully explain human aesthetic responses to flowers. We discuss alternative explanations. This topic deserves renewed attention from researchers working in related fields.

## Graphical abstract

**Figure:**
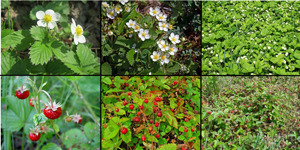

**Media summary:** Contrary to the assumptions of the habitat selection theory, flowers elicit stronger aesthetic responses than fruits.

## 1. Background

Human attraction towards flowers is a phenomenon that is manifested in various ways, from ornamental gardens and flower exhibits to product design and get-well gifts. The volume of the global cut flower trade reaches €15 billion per year (Mamias, 2018). One can find abundant examples of the aesthetic appreciation of flowers in many different cultures and historical periods. Some authors consider the tendency to aesthetically appreciate plants and flowers as probably common to humans as a species and related to biotope choice (Appleton, 1996; Eibl-Eibesfeldt, 1989; Kellert, 1995; Wilson, 1984). One might ask why this behaviour evolved.

*Terminological note:* In this paper, following Renoult (2016), we call the mental process by which a rater decides where to place a stimulus on a rating scale from ugly to beautiful an *aesthetic evaluation*. We use the term *aesthetic preference* or just *preference* when a rater compares the attractiveness of two stimuli in terms of their beauty. By *aesthetic response*, we mean the result of an aesthetic evaluation (for example, the score a rater gave to a stimulus on a rating scale). Berlyne (1971) and Ulrich (1983, 1986) described the term *aesthetic response* as a preference or a like–dislike effect in association with pleasurable feelings caused by visual exposure to a stimulus, i.e. a focus predominantly on emotions and affects. Our definition is broader because the aesthetic evaluation that precedes the aesthetic response also includes perception and cognition (Redies, 2015; Renoult & Mendelson, 2019). This definition also corresponds well with the habitat selection theory (described in detail later in the paper). This theory states that environmental stimuli trigger perceptual, cognitive and emotional processes that lead to adaptive responses (Orians & Heerwagen, 1992). Although measurements of the aesthetic response might be performed using different scales (such as like–dislike or beautiful–ugly), such choices do not lead to significantly different results; the scales are highly correlated (Ulrich, 1986; Zube et al., 1975).

Some of the proponents of evolutionary aesthetics link the aesthetic response to function and adaptive value (Voland & Grammer, 2003): our ancestors evolved to consider environments, objects or situations that increased their chance of survival and reproductive success as beautiful and those that decreased it as ugly (Ruso et al., 2003; Thornhill, 2003; Ulrich, 1986). Therefore, emotional responses to beauty and ugliness should represent an adaptive reaction to potentially beneficial or harmful situations because they are very rapid and strong motivators of human behaviour (Heerwagen & Orians, 1995).

The emotional response is stronger for signals that are more important for the receiver (Ulrich, 1983). This is evident, for example, in the Open Affective Standardized Image Set database (Kurdi et al., 2017), where images differ in their valence (positive vs negative) and arousal (low vs high). Images showing imminent threats on the one hand and good foraging opportunities and safe spots on the other are highly arousing. Highly arousing positive images depict, for example, landscapes with water, vegetation and vistas; lowly arousing include images of lawns or monotonous fields. Highly arousing images with negative valence include wildfires or severe drought, whereas lowly arousing ones might display junk or rubbish yards.

Judith Heerwagen and Gordon Orians applied the adaptive approach to evolutionary aesthetics in their theory of habitat selection (Heerwagen & Orians, 1995; Orians & Heerwagen, 1992). The theory incorporates environmental cues crucial for our ancestors’ survival – such as the presence of food, fresh water and shelter, and the ability to easily orient themselves in the landscape to avoid predators and other threats – into one conceptual framework.

The theory describes spatial and temporal frames of habitat selection. Different aspects of habitat are central for each of these frames. The spatial frame includes decisions on whether and how profoundly to explore an area and whether it is suitable for various activities. The temporal frame distinguishes environmental cues that require immediate attention (e.g. an incoming storm), cues associated with seasonal changes (e.g. the leafing out of trees) and cues influencing long-term behaviour (e.g. the presence of a lake). The authors also argue that because a suitable habitat has to fulfil the needs of many different activities across time, people evolved not only to evaluate the immediate state of the environment, but also to pay attention to features that might help them to predict its probable future states.

Flowers are a typical cue associated with seasonal change. They signal important, positive changes in resource availability and represent a promise of good foraging opportunities in the future. Thanks to their specific appearance, they also help people to distinguish and localise different types of resources. Thus, paying attention to flowers had adaptive value because it improved human functioning in natural environments (Orians & Heerwagen, 1992).

In their latter work, the authors emphasise a more direct link between flowers and food. They describe flowers as a potential food source, rich in nitrogen compounds and relatively free from toxins (compared with other plant parts). Furthermore, bees use pollen from flowers to produce honey, which has long been a highly appreciated natural sugar source (Heerwagen & Orians, 1995). The authors also speculate about a possible human preference for flowers with zygomorphic or otherwise unusual shapes, because on average, they contain more nectar and pollen. However, a more recent empirical study found the opposite: raters disliked zygomorphic and unusual flowers (Hůla & Flegr, 2016).

Heerwagen and Orians state that conceptual theories about human responses to flowers are lacking and that the habitat selection theory offers a potentially powerful approach to this issue (Orians & Heerwagen, 1992).

Although the habitat selection theory offers testable hypotheses and was formulated almost 30 years ago, it has not yet been empirically tested in relation to human aesthetic responses to flowers. This might be due to the fact that the topic of human perception of flowers has long been entirely out of the scope of evolutionary aestheticians and other researchers from related fields (which also explains the lack of current literature in the theoretical part of this paper). However, in recent years there has been renewed interest in the study of the aesthetics of flowers and related human–plant interactions centred on plant morphology (Elsner & Wertz, 2019; Hůla & Flegr, 2016; Oberzaucher, 2017; Wertz & Wynn, 2014a, 2014b; Włodarczyk et al., 2018). There has also been a recent call for theory- and hypothesis-driven research in ethnobotany (Gaoue et al., 2017) with an emphasis on the integration of an evolutionary approach (de Albuquerque & Hanazaki, 2009). This leads us to believe that it is necessary to further explore the proposed theoretical framework of Heerwagen and Orains as it might be hugely beneficial for the whole field if supported by empirical data.

As we described above, Heerwagen and Orians suggest that food and resource availability cues trigger aesthetic responses in humans. They argue that flowers represent such a cue, and that is why humans like them. We decided to follow the habitat selection theory's logic and compared two types of stimuli related to seasonal change and resource availability – flowers and fruits. Both cues have a positive valence, but they differ in their importance for the receiver. In contrast to fruits, flowers are only exceptionally eaten by humans and seldom by other large African primates. In contrast, fruits are among the most important food sources (Heymann, 2011; Marlowe & Berbesque, 2009; Newton-Fisher, 1999; Peters et al., 1981, 1984). Fruits also usually contain large amounts of sugar and are generally nutritionally richer than flowers. On the other hand, flowers represent a fallback food for some primates (Heymann, 2011; Hogan et al., 2016), including some populations of chimpanzees (Newton-Fisher, 1999). However, research on Hadza hunter–gatherers showed that even an essential fallback food (tubers) was the least preferred of all food types (Marlowe & Berbesque, 2009).

Flowers are ephemeral when compared with fruits. A flower blooming for a week is considered to be long-lived. A majority of flowers blooming during the day in a hot or dry climate, for example, in the African savanna, do not last more than a single day (Primack, 1985). Flowers thus represent an approximation of possible and uncertain future resource availability. In contrast, fruits are an instant, direct and strong signal of the presence of resources at a given moment and, thanks to their relatively greater longevity, in the near future (weeks). This leads us to the conclusion that, if the habitat selection theory is correct, fruits should elicit stronger responses and should be preferred more than flowers.

Surprisingly, Heerwagen and Orians do not pay much attention to fruits. They only mention ripening fruits as a positive cue related to seasonal change (Orians & Heerwagen, 1992). This might be due to the fact that the relationship between fruits and food is simple and straightforward and does not require a particular explanation in their eyes.

## 2. Hypothesis

Our main objective was to determine if there is empirical evidence for the theory of human aesthetic responses to flowers proposed by Gordon Orians and Judith Heerwagen (Heerwagen & Orians, 1995; Orians & Heerwagen, 1992).

We formulated a testable hypothesis:

Plant species with edible fruits will receive a higher score on the rating scale (from very ugly to very beautiful) during the fruiting stage than during the flowering stage.

### 2.1. Exploratory part

In the exploratory part of the study, we first wanted to examine whether women and men differ in their ratings of flowers and fruits. Some authors have speculated that there might be sex differences in the aesthetic preferences of natural environments owing to the hypothetically predominant role of women as gatherers and men as hunters in human evolutionary history (Ruso et al., 2003). This differentiation of roles between men and women already serves to explain sex differences in other aspects of human behaviour and abilities, such as orientation in space – the so-called hunter–gatherer theory of spatial sex differences (Silverman et al., 2007; Silverman & Eals, 1992).

A considerable number of studies have found differences in general human colour preferences (reviewed in Crozier, 1999) and have discussed possible explanations for these differences (Hurlbert & Ling, 2007; Palmer & Schloss, 2010; Sorokowski et al., 2014). There is also evidence for differences in the colour preferences of flowers (Hůla & Flegr, 2016; Yue & Behe, 2010) and trees (Muderrisoglu et al., 2009). For these reasons, we wanted to explore whether the colour of flowers and fruits in our dataset influenced their rating.

## 3. Materials and methods

The Charles University review board approved this research (approval no. 2017/10).

### 3.1. Study A – Czech plants

#### Stimuli

In study A, we used photographs of 14 plant species with edible fruits native to or commonly cultivated in the Czech Republic. The discussed theory emphasises the importance of fruits and flowers in relation to food resources, so we wanted the raters to know that the fruits were indeed edible. There were four herbs, five shrubs and five trees in the set (see Table 1). Each species was displayed in the flowering stage and the fruiting stage and from three different distances: 1, a close-up of the flowers/fruits; 2, flowers/fruits with a part of the plant (photographs were taken from a distance of 0.5–1 m); and 3, the whole plant with flowers/fruits. In total, there were six photographs per species. By using the same species for displaying both flowers and fruits, we tried to minimise a possible bias that might occur if we displayed flowers and fruits of entirely different plants (different leaf shapes, the habitus of the plant, etc.). We also tried to choose a wide range of flower and fruit colours.

**Table 1.** A list of stimuli used in study A (Czech plants) and studies B and C (African plants)

Scientific name	Common name	Habitus	Flower colour	Flower colour group	Fruit colour	Fruit colour group
**Czech plants**						
*Castanea sativa*	Sweet chestnut	Tree	Yellow	Yellow	Green	NA
*Citrus sinensis*	Sweet orange	Tree	White	White	Orange	NA
*Corylus avellana*	Common hazel	Tree	Yellow	Yellow	Green	NA
*Cornus mas*	Cornelian cherry	Tree	Yellow	Yellow	Red	Red
*Cucurbita pepo*	Pumpkin	Herbaceous	Yellow	Yellow	Orange	NA
*Fragaria vesca*	Wild strawberry	Herbaceous	White	White	Red	Red
*Pisum sativum*	Pea	Herbaceous	White	White	Green	NA
*Prunus domestica*	Plum	Tree	Pink	NA	Yellow	Yellow
*Prunus spinosa*	Blackthorn	Shrub	White	White	Blue	Blue/black
*Ribes petraeum*	Rock currant	Shrub	Pink	NA	Red	Red
*Rubus fruticosus*	Blackberry	Shrub	White	White	Black	Blue/black
*Sambucus nigra*	Elder	Shrub	White	White	Black	Blue/black
*Solanum lycopersicum*	Tomato	Herbaceous	Yellow	Yellow	Red	Red
*Vaccinium myrtillus*	Blueberry	Shrub	Pink	NA	Blue	Blue/black
**African plants**						
*Abelmoschus esculentus*	Okra	Herbaceous	White, Yellow	White/Cream	Red, Brown	Red/Brown
*Adansonia digitata*	Baobab	Tree	White, Yellow	White/Cream	Green	Green
*Annona senegalensis*	African custard apple	Shrub	White, Yellow	White/Cream	Yellow, Brown	Brown/Yellow
*Blighia sapida*	Ackee apple	Tree	Yellow	Yellow	Red, Brown	Red/Brown
*Calodendrum capense*	Cape chestnut	Tree	White, Pink	NA	Green	Green
*Carissa macrocarpa*	Natal plum	Shrub	White	White/Cream	Red	Red/Pink
*Combretum erythrophyllum*	River bushwillow	Tree	Red	Red/pink	Red, Brown	Red/brown
*Diospyros lycioides*	Bushveld bluebush	Shrub	Yellow	Yellow	Red, Brown	Red/brown
*Dovyalis caffra*	Kei apple	Tree	Yellow	Yellow	Yellow	Yellow
*Euclea racemosa*	Sea guarrie	Tree	White, Brown	Brown/yellow	Blue	NA
*Garcinia livingstonei*	African mangosteen	Tree	Yellow, Green	NA	Orange	NA
*Grewia flava*	Grewia	Shrub	Yellow	Yellow	Red, Brown	Red/brown
*Kigelia africana*	Sausage tree	Tree	Red, Brown	Red/brown	White, Brown	White/cream
*Lagenaria siceraria*	Calabash	Herbaceous	White	White/cream	Green	Green
*Momordica balsamina*	Balsam apple	Herbaceous	Yellow	Yellow	Orange	NA
*Parkia biglobosa*	African locust bean	Tree	Red	Red/pink	Green	Green
*Passiflora edulis*	Passion fruit	Herbaceous	Violet, white	NA	Violet, Brown	Red/brown
*Syzygium cordatum*	Water berry	Tree	Pink	Red/pink	Pink, Violet	Red/pink
*Tamarindus indica*	Tamarind	Tree	Pink, yellow	NA	Brown	Brown/yellow
*Vachellia tortilis*	Umbrella thorn	Tree	Yellow	yellow	Brown, Green	Brown/yellow

Note: flower/fruit colour = the colour(s) of a given species’ flowers/fruits; flower/fruit colour group = the colour group to which we assigned a given species for the purpose of ANOVA.

**Figure 1.**
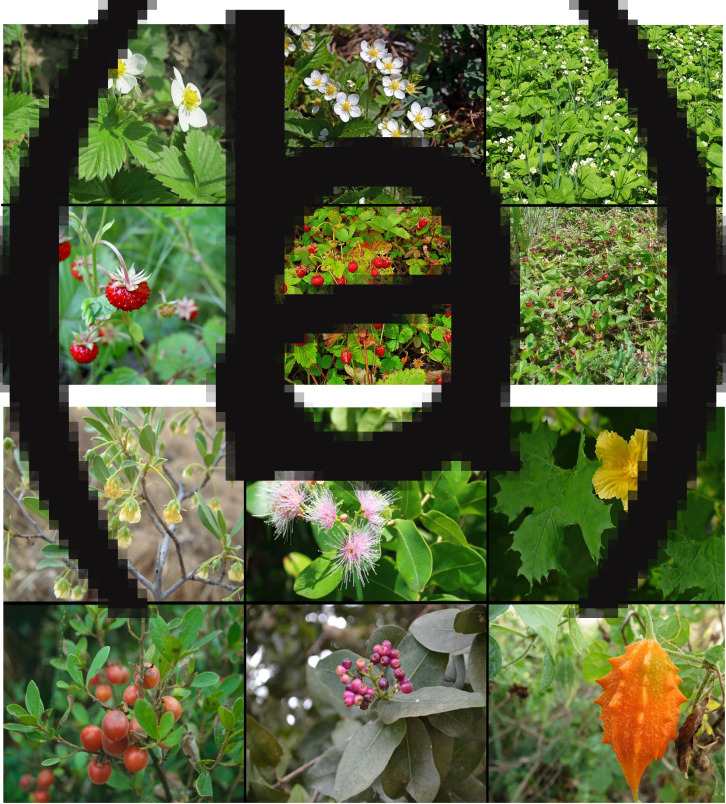
Examples of stimuli. Note: (a) wild strawberry (*Fragaria vesca*) used in Study A. Left *=* close-up, centre = 0.5–1 m, right = whole plant, top = flowering, bottom = fruiting. (b) Examples of stimuli used in Study B. Left = Bushveld bluebrush (*Diospyros lycioides*), centre = water berry (*Syzygium cordatum*), right = balsam apple (*Momordica balsamina*), top = flowering, bottom = fruiting. (a) The photographs are public domain (CC0) except for bottom centre: ‘Fragaria vesca 003.JPG’ by H. Zell, licensed under CC BY-SA 3.0, and bottom right: ‘Jahodník obecný’, photo courtesy of Planta Naturalis. (b) From top left: ‘Diospyros lycioides Desf.’ by S. Rügheimer et al., licensed under CC BY-NC; ‘Syzygium cordatum Hochst. Ex O. Krauss’ by P. Horn, licensed under CC BY-NC; ‘Momordica balsamina 002.JPG’ by H. Zell, licensed under CC BY-SA 3.0; ‘Diospyros lycioides’, and ‘Syzygium cordatum’, photos courtesy of Random Harvest Nursery; ‘Momordica charantia, fruit’ by Katja Schulz, licensed under CC BY 2.0.

We used non-standardised freely available photographs from the internet. In a few cases, we used private photographs. Their owners gave us written permissions to use the photographs for this research. We tried to control for the plant–background ratio and also rescaled the images to the same size (600 × 450 px), see Figure 1. Data from an independent study show that images from the internet can be used as a substitute for real flowers or standardised images in preference ratings. These results were already presented to an international audience (Hůla et al., 2018), but have not yet been published. We also asked 12 independent raters to indicate the correct distance for each photograph. We then replaced a few problematic photographs in cases where one of the raters judged the distance incorrectly.

All stimuli are available from https://figshare.com/s/e124d9ad5a57bde18ce7

#### Display

The rating of the photographs was part of a broader anonymous online questionnaire that consisted of several unrelated topics. We created the questionnaire using Qualtrics software. In the part relevant to this study, participants first answered basic demographic questions. They also specified whether they had any sight conditions, such as colour blindness. During the rating, there was displayed a photograph of a plant and a question, ‘How do you like the plant in the photograph?’ Participants then chose a number on a six-point scale where 1 meant ‘it is very ugly’ and 6 meant ‘it is very beautiful’. We instructed the participants to rate only how they liked the plant itself, not the composition or quality of the photograph. Each rater was randomly assigned to one distance and rated 28 photographs (14 species, two growth stages) in random order.

### 3.2. Participants

The questionnaire was in Czech and aimed at the Czech (and partly Slovak) population. However, participation was open to anybody who understood Czech. The majority of participants came from the *Lab bunnies* [Pokusní králíci] community grouped around the Facebook and web pages administered by our team. Lab bunnies consist of more than 20,000 Czech and Slovak volunteers willing to participate in evolutionary psychology experiments. We recruited the participants using a Facebook-based snowball method. Any participant could share the link to the questionnaire. Before proceeding to the questionnaire, each participant had to read information about the research and consent to take part in it. There were no restrictions on participation.

We excluded data from colour-blind participants as well as from all participants who showed no variance in ratings, rated fewer than 24 out of 28 photographs, or failed on both questions testing their attention, e.g. ‘Please check number 2 on the scale’. Nine-hundred and seventeen participants (mean age = 35.02 ± 12.80; female = 591, male = 326) rated the close-up photographs, 921 participants (mean age = 34.31 ± 12.52; female = 630, male = 291) rated the photographs taken from 0.5 to 1 m and 954 participants (mean age = 34.27 ± 12.58; female = 625, male = 329) rated the photographs of the whole plant.

### 3.3. Study B – plants of the African savannas

Study B's objective was to repeat study A with an independent set of stimuli and raters. Ten months after starting the online questionnaire data collection, we replaced the photographs used in study A with a new set of stimuli. The new set contained 20 plant species with edible fruits native to the African savannas (Table 1). There were only close-up images, so each participant rated 40 photographs (20 species, two growth stages). Otherwise, the setting was identical to that of Study A.

We used only close-up photographs because we found no distance-related differences in the rating of the flowering and fruiting stages in study A (see Section 4.1 of Results). We also decided to use species generally unknown in the Czech Republic so that the participants could not connect the images with the taste of the fruits or possible emotional personal memories related to the displayed plants. Since the authors of the habitat selection theory operate on the presumption that human landscape and habitat preferences were shaped in African savannas, we decided to use species native to this biome.

The stimuli are available from https://figshare.com/s/3c7cb1fa138b8fab6973

We obtained data from 743 participants (mean age = 34.3 ± 13.36, female = 457, male = 286). As in Study A, we excluded data from colour-blind participants as well as from all participants who showed no variance in ratings, rated fewer than 36 out of 40 photographs or failed on both questions testing their attention.

### 3.4. Study C – African plants: two-alternative forced-choice method

Since the rating paradigm might dramatically impact the results, as shown, for example, in Jones and Jaeger (2019), we decided to conduct a third additional study. We used the stimuli from study B (close-ups of the plants of African savannas). However, this time, the participants did not perform aesthetic evaluations using rating scales but expressed their preference in a two-alternative forced-choice paradigm.

The photographs were displayed in pairs placed horizontally next to each other. Each pair consisted of the same plant species in the flowering and fruiting growth stage. There were 20 pairs in total. The participants answered a question: ‘Which plant do you like more?’ by clicking on a preferred photograph. Again, we instructed the participants to express only how they liked the plant itself, not the photograph's composition or quality.

For each participant, we randomised the display order of the pairs of photographs and the position of the photographs within each pair.

817 participants (mean age = 36.6 ± 11.6, female = 570, male = 247) completed the questionnaire. We excluded participants who rated fewer than 18 out of 20 pairs. However, there were only seven cases with any missing values in our final dataset.

### 3.5. Statistical analysis

We used R 3.5.1 (R Core Team, 2019) and RStudio 1.1.463 (RStudio Team, 2019) for the statistical analyses and ggplot2 (Wickham, 2016) and ggstatsplot (Patil, 2018) packages for the graphs. We set the alpha level for all statistical tests to 0.05.

To test the hypothesis that fruiting plants score higher than flowering plants in the aesthetic evaluation, we compared each participant's mean rating for fruiting plants with that for flowering plants (Studies A and B). Because each participant rated the same species in two growth stages, we used a two-sided paired *t*-test for the comparison. We ran the test separately for each distance.

In Study C, we calculated the proportion of preferred fruiting plants for each participant. A value of 0 meant the absolute preference for flowering plants and 1 the absolute preference for fruiting plants. We used a single sample *t*-test to compare this proportion with the assumption that there was no difference between the preference for fruiting and flowering plants (the proportion equals 0.5).

We used a pwr package (Champely, 2018) for power analysis. The sample size necessary for the determination of an effect size of 0.15 with a power of 0.9 equalled 469 in all studies (A + B, two-sided paired *t*-tests; C, two-sided single sample *t*-test). Since our research was part of a broader online survey, we waited until the survey termination and analysed all obtained data, which is why our sample size exceeded the requirements of power analysis in Studies A and B. We conducted Study C later, as an independent survey. We terminated it when we obtained a similar sample size to that in the previous two studies. We used the Holm–Bonferroni method for the correction of multiple tests.

In the exploratory part of the study, we used two-way ANOVAs to determine whether there were any sex differences in the rating of fruits and flowers. We also used Welch's ANOVA and a subsequent Games–Howell *post hoc* test to explore the possible influence of differently coloured fruits and flowers on the preference ratings.

## 4. Results

### 4.1. Study A – Czech plants

**Figure 2.**
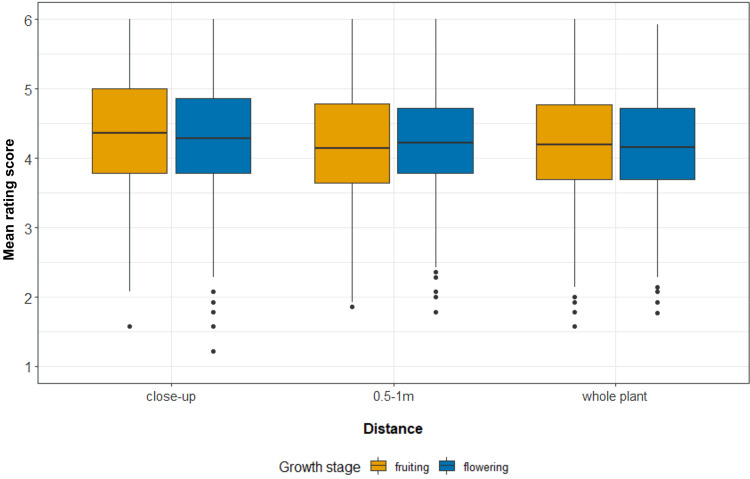
Differences between the aesthetic response to fruiting and flowering Czech plants. Note: *x*-axis, distance; *y*-axis, mean rating score (in points) of all plants in fruiting (orange) and flowering (blue) growth stages. The whiskers represent 1.5 interquartile range (IQR).

**Figure 3.**
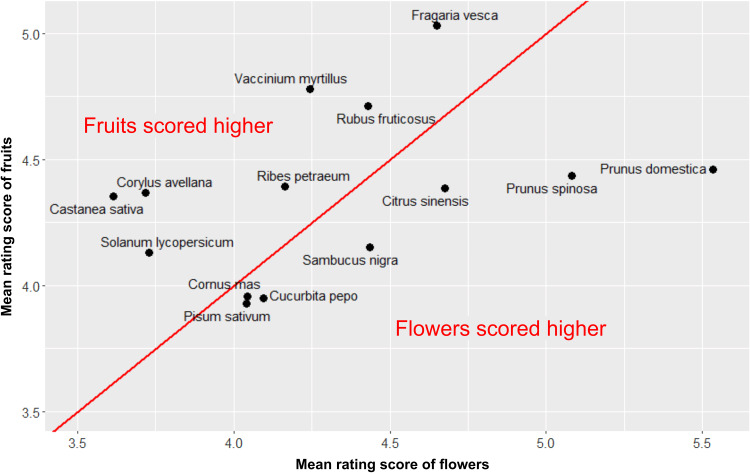
Scatterplot of the aesthetic response to the close-up photographs of Czech plants. Note: *x*-axis, the mean rating score of the flowering stage; *y*-axis, the mean rating score of the fruiting stage. The red line represents values where the rating scores for both growth stages are the same.

In the close-ups, the participants liked the photographs of fruits more than the photographs of flowers (Figure 2; *t* = 2.20, d.f. = 916, *p*-value = 0.028, mean difference = 0.042 points, 95% CI [0.048, 0.084], Cohen's *d* = 0.073). We found higher ratings for flowers at the distance of 1 m (mean difference = −0.033 points, 95% CI [−0.066, –0.0015], *t* = −2.05, d.f. = 920, *p*-value = 0.040, Cohen's *d* = 0.068). There was no difference between the ratings of fruits and flowers at the ‘whole plant’ distance (mean difference = 0.019 points, 95% CI [−0.0059, 0.044], *t =* 1.49, d.f. = 953, *p*-value = 0.14, Cohen's *d* = 0.048). However, the effect sizes were very small. After correcting for multiple tests, no statistically significant differences remained. The distribution of species by the rating of their flowering or fruiting stage (only in the close-up photographs) is shown in Figure 3.

#### Study A – exploratory part

We used a two-way ANOVA to determine whether women and men differed in their ratings of flowers and fruits. Each rater's mean difference between the rating of flowering and fruiting plants represented the dependent variable. The rater's gender (man, woman) and the photographic distance displayed to the rater (close-up, 0.5–1 m, whole plant) represented the factors.

Overall, the ANOVA model revealed a significant interaction between distance and gender, but no effect of gender *per se*. The subsequent summary model (*F*_5, 2786_ = 3.52, *p*-value < 0.0036, *R*^2^ = 0.0062) showed that the only difference between men and women occurred at the ‘whole-plant’ distance, where men liked fruits slightly more than women. However, the difference was negligible (mean difference women = 0.01 points, mean difference men = 0.05 points; the maximum possible difference was 5 points).

To study the possible influence of colour on the aesthetic evaluation of flowers and fruits in our sample, we used one-way ANOVA. The mean rating for both fruiting and flowering plant species (from all respondents) served as the dependent variable, and the fruit and flower colours were the factors. This means that we analysed both fruiting and flowering plants together (analysed colours, blue/black, red, white, and yellow. We excluded green, orange and pink fruits and flowers from the analysis because of their low number; see Table 1). We performed the testing separately for each distance. Owing to the low number of observations per group (four to six) and their unequal variances at all distances, we used Welch's ANOVAs. We did not find any statistically significant differences among colours at any of the distances. However, yellow colour had the lowest mean rating at all distances.

### 4.2. Study B – plants of the African savannas

**Figure 4.**
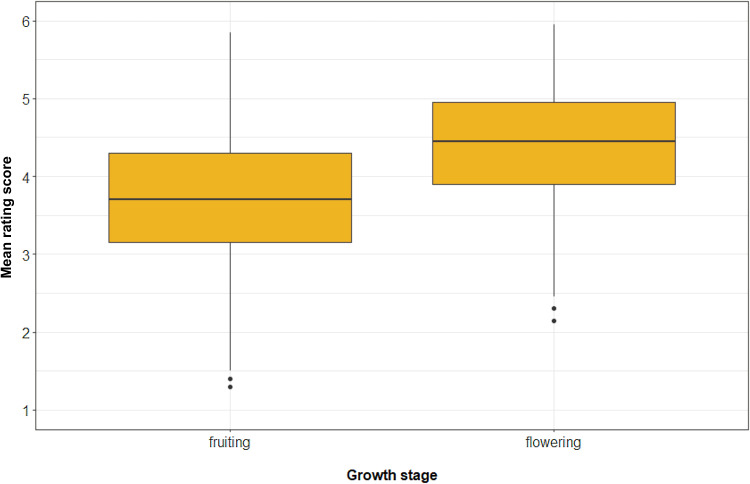
Differences between the aesthetic response to fruiting and flowering African plants. Note: *x*-axis, growth stage (fruiting, flowering); *y*-axis, mean rating score (in points) of all plants. The whiskers represent 1.5 IQR.

**Figure 5.**
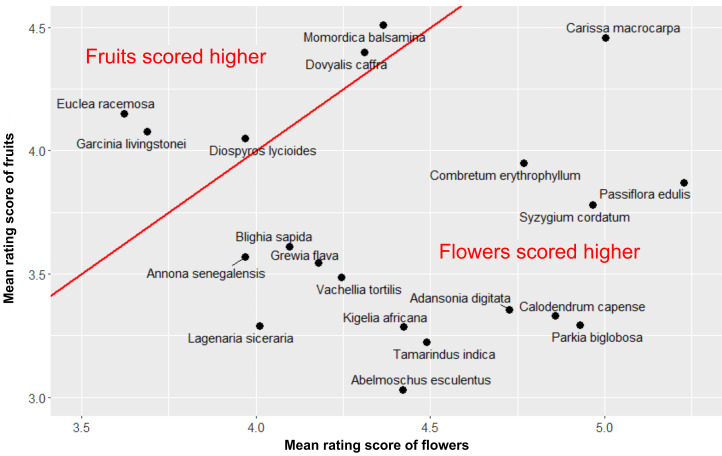
Scatterplot of the aesthetic response to the close-up photographs of African plants. Note: *x*-axis, the mean rating score of the flowering stage; *y*-axis, the mean rating score of the fruiting stage. The red line represents values where the aesthetic response to both growth stages is the same.

As we explained above, the participants rated only the close-up photographs of plants in this study. The difference in the ratings between the flowering and fruiting plants was clearly in the opposite direction than what we hypothesised. The two-sided paired *t*-test confirmed that flowering plants were liked more than fruiting plants and that the effect size was very large (*t =* −33.94, d.f. = 743, *p*-value < 0.0001, mean difference = −0.70 points, Cohen's *d* = 1.24; see Figure 4). The distribution of species by the rating of their flowering or fruiting stage is shown in Figure 5.

#### Study B – exploratory part

To explore possible differences between the ratings of women and men, we used an independent samples two-sided *t*-test. Each rater's mean difference between the rating of fruiting and flowering plants represented the dependent variable, and the rater's gender was the factor. We found that both women and men gave flowering plants higher ratings, but this difference was more pronounced in women (mean difference women = −0.77 points, mean difference men = −0.58 points, *t =* −4.58, d.f. = 741, *p* < 0.0001, Cohen's *d* = 0.35).

**Figure 6.**
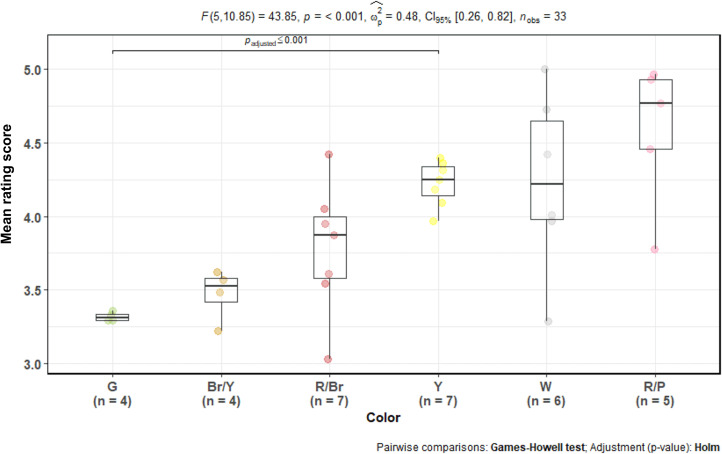
Aesthetic response to African plants by colour. Note: one-way Welch's ANOVA of mean rating scores of African plants with different flower/fruit colours. *x*-axis, colour groups (G = green, Br/Y = brownish yellow, R/Br = brownish red, Y = bright yellow, W = white or cream, R/P = red or pink). *y*-axis, mean rating score of all plants (in points). The coloured dots represent each plant species (flowering and fruiting stages of the same species are represented as separate dots).

To test the effect of colours on aesthetic evaluations, we used a one-way Welch's ANOVA. The reason for this decision was that the number of observations per group was not balanced, and the groups had substantially different variances. We used the Games–Howell *post hoc* test to compare the differences between groups. The analysed flowers and fruits rarely had a basic colour, so we identified several colour groups: green, reddish-brown, brown/yellow, red/pink, white/cream, yellow. We excluded seven photographs from the analysis because of their unique colour or colour combination. The model showed that there were statistically significant differences between colours (*F*_5, 10.85_ = 43.85, *p*-value < 0.0001, *ω*^2^ = 0.48). When corrected for multiple comparisons by Holm's method, the Games–Howell test found a significant difference only between green and yellow colours, where yellow had a higher rating. However, a clear trend showed that green and brownish yellow were among the least liked colours and pink and pure red among the most liked (Figure 6).

### 4.3. African plants: two-alternative forced-choice method

The single sample *t*-test showed that the proportion of preferred fruiting plants was significantly lower than the theoretical assumption of 0.5. The effect size was very large (mean proportion of preferred fruiting plants = 0.36, 95% CI [0.35, 0.37], *t =* −23.885, d.f. = 816, *p* < 0.0001, Cohen's *d* = 0.84). Participants in our dataset thus strongly preferred flowering plants over fruiting plants of the same species.

## 5. Discussion

We have divided the discussion into several sections. First, we briefly summarise the most important results, then we discuss the possible effect of the raters’ familiarity and knowledge of the presented plants, a possible effect of the different colours and shapes of the stimuli, and cultural influences. In the general discussion, we place our findings into a broader theoretical context.

### 5.1. Summary of results

Contrary to the assumptions we derived from the simple variant of the habitat selection theory proposed by Heerwagen and Orians, respondents rated Czech fruits and flowers as almost equally attractive. The lack of any observed difference between flowers and fruits is not surprising for the whole plant photographs. In some cases, it might be problematic to distinguish flowers from fruits from such a distance. In contrast, at the other two distances (close-ups and 0.5–1 m), flowers and fruits were easily recognisable.

In Study B (African plants), participants rated fruits as much less attractive than flowers. The mean difference was equal to 0.70 points, and the effect size was very large (Cohen's *d* = 1.24).

In Study C (African plants – forced-choice), we confirmed the results from Study B on an independent set of raters and using a different display paradigm that tested explicitly for preference (choosing what one likes more). We observed a strong preference for the flowering stage (Cohen's *d* = 0.84).

The absolute rating scores of flowering and fruiting plants across Studies A and B are worth a look. We can see that the mean ratings of flowering plants were very similar for both Czech and African plants (4.30 and 4.41, respectively) but differed for fruiting plants (4.34 and 3.71, respectively; see also Figures 2 and 4). The mean rating of fruiting plants was much lower in the case of African species. Of course, one has to be cautious in drawing any conclusions from such a comparison. Although the display paradigm and rating scales were identical in both studies, they differed in stimuli, raters and the ratings’ variance. On the other hand, the difference in variances was still acceptable (Study A – close-ups: var flowers = 0.91, var fruits = 0.89; Study B: var flowers = 0.64, var fruits = 0.67).

### 5.2. A possible effect of familiarity and knowledge of plants

#### Edibility

The stimuli in Study A represented edible fruits commonly grown or known in the Czech Republic. Although we did not explicitly ask the raters about their familiarity with each of the displayed plants, we can assume that the vast majority of participants knew that the displayed fruits were safe to eat and tasty. However, although all African species displayed in Study B (and C) had edible fruits, they were almost certainly unknown to most of the Czech raters. A study conducted on the geographically and culturally very close Slovak population found that children could not distinguish unknown fruits’ toxicity and that this inability did not improve with age (Prokop & Fančovičová, 2014). Such uncertainty about the edibility of African fruits could have accounted for their lower rating.

#### Familiarity

The respondents’ general familiarity with the stimuli might influence their ratings. Many studies have reported a positive correlation between familiarity and the aesthetic responses to various objects (for review, see, for example, Bornstein, 1989). Some studies also found this effect on environmental preference (Balling & Falk, 1982; Pedersen, 1978). However, more recent research suggests that the preference for familiarity differs across object categories. For example, Park et al. (2010) performed a series of experiments and found a preference for familiarity in faces, a preference for novelty in natural objects and scenes (including flowers), and no preference related to familiarity or novelty in geometric figures. It is thus uncertain whether familiar or unknown flowers and fruits should be preferred. Nevertheless, both flowers and fruits in Study B (African plants) were most probably unknown to the raters. Hence, familiarity could not account for the different ratings of African flowers and fruits. However, it might influence the ratings of the Czech sample. We can assume that Czech people easily identified the displayed fruits because they commonly buy and eat them. The raters’ familiarity with the plants’ flowering stages was probably lower since not everyone is interested in gardening or walks in nature.

#### Odour

Odours associated with the stimuli might also play some role in the ratings. Scents and pictures seem to exhibit interactive effects on sensory imagery (Lwin et al., 2010). For example, Koubaa and Eleuch (2020) showed that visually induced olfactory imageries influence taste perception and food consumption.

Flowers are usually associated with pleasant odours, which could influence the raters. On the other hand, the results of the studies mentioned above were obtained by working with specific scents that could be easily attributed to their source (such as rose and lavender in the case of flowers, vanilla, and chocolate of other stimuli). It would be challenging to associate most of the flowering plants in our research with their specific scents. We remind that the African species were almost certainly unknown to the raters. In the Czech plant sample, only two species had fragrant flowers (*Citrus sinensis* and *Sambucus nigra*). However, many of the fruits in the Czech sample (if not all) had a distinct and pleasant scent, known to the raters (such as blueberries, strawberries, plums and oranges). Thus, we can assume that if there was any effect of olfactory imagery on the ratings, it was probably in favour of fruits in Study A. It is questionable whether a general assumption that ‘flowers smell nice’ might affect the unknown plants’ ratings in Studies B and C.

#### Prototypicality

In general, people prefer high levels of prototypicality, which is a measure of how representative an object is of a category (Reber et al., 2004; Whitfield & Slatter, 1979; Winkielman et al., 2006). It is difficult to objectively compare the levels of prototypicality of flowers and fruits. Both flowering and fruiting plants in our samples probably contained species with various levels of prototypicality.

### 5.3. A possible effect of colour

Another factor that could possibly influence the stimuli's rating was the colour of the flowers and fruits. Even though we tried to assure an equal distribution of the same colours between fruits and flowers, we were severely limited by the fruits’ required edibility. This resulted in a relatively higher distribution of blue and red among fruits and yellow and white among flowers in our Czech stimuli set. The colours were more evenly distributed in African plants, but there were more brownish colours among African fruits (see Table 1). Previous research on flowers found that blue was the most and yellow the least liked flower colour (Hůla & Flegr, 2016). Research conducted on Slovak high school students revealed that red fruits were considered edible and attracted the most attention of the raters (Prokop & Fančovičová, 2012). Also, blue and red have been rated as the most and brown and yellow as the least appealing colours in general (Camgöz et al., 2002; Palmer & Schloss, 2010). Therefore, we can assume that if the colour of a stimulus influenced its rating, it should have been in favour of fruits in the case of Czech plants because they had a higher proportion of generally appealing colours. However, when we performed ANOVA to distinguish whether there were any differences in the ratings of differently coloured stimuli, we found no significant difference between colour groups in the Czech sample.

In the case of African plants, there was a difference between green stimuli (all of which were fruits) and yellow stimuli (predominantly flowers); the green stimuli had the lowest mean rating (see Figure 6). This difference between green and yellow stimuli is also interesting. As we already discussed in various parts of this paper, yellow is considered one of the least favourite colours in general and one of the least liked flower colours. This might mean that the observed difference is not due to the stimuli's colour but rather due to their type (flower vs fruit). Moreover, previous research found that in the case of flowers, colour played only a minor role in their overall attractiveness; shape properties were the most important (Hůla & Flegr, 2016).

### 5.4. A possible effect of shape properties

Some literature suggests that in general, people prefer shapes with several axes of symmetry (Tinio & Leder, 2009), round contours (Bar & Neta, 2006, 2007; Leder et al., 2011; Silvia & Barona, 2009; Westerman et al., 2012) and medium levels of complexity (Akalin et al., 2009; Enquist & Johnstone, 1997; Reber et al., 2004). As with prototypicality mentioned above, it is difficult to compare the levels of complexity across flowers and fruits objectively. However, in both our samples, the fruits were usually rounder and had more axes of symmetry than the flowers.

### 5.5. Cultural influences

Habitat selection theory considers the human aesthetic appreciation of flowers to be adaptive. However, some authors, such as Jack Goody (1993), try to explain human aesthetic responses to flowers by a combination of cultural and environmental factors.

According to Goody, extensive aesthetic interest in flowers appeared with the dawn of advanced agriculture, which brought the possibility to accumulate and store food surpluses and led to the formation of highly stratified societies. The people at the top of the societal hierarchy had enough food and wealth to pursue new, non-utilitarian activities and to develop a ‘culture of luxury’. Flowers started to be domesticated, cultivated in ornamental gardens and used as luxury items for the rich, as motifs in visual arts, and during various ritual ceremonies (pp. 18, 415). However, Goody distinguishes the complex aesthetic appreciation of cultivated flowers from a simple aesthetic interest in wildflowers, the former being a product of advanced agriculture, while the latter not (p. 20).

Goody is especially intrigued by the striking lack of interest in both wild and cultivated flowers among Sub-Saharan African cultures. He points out that these societies are very socially homogenous and often practise simple hoe agriculture. However, this would only explain their lack of interest in cultivated flowers. For this reason, Goody further hypothesises that environmental factors play a role, namely the somewhat surprising absence of wildflowers in Africa's forests and savannas (pp. 13–14, 19). An environmental explanation for different cultural attitudes towards flowers was also proposed, for example, by Komárek (2009).

Nevertheless, Goody soon adds that even environmental factors are not a sufficient explanation. For example, the Barasana people from the flower-rich Amazon region in South America do not like wildflowers. He proposes that, in some cases, people deliberately rejected flowers, such as for religious reasons, but he is not sure whether this also occurred in Sub-Saharan Africa (pp. 20, 24).

What Goody describes as a *total* lack of interest in flowers is rather a *relative* lack of interest. In other words, Goody describes quantitative differences in the aesthetic interest in flowers, not qualitative differences. This is nicely illustrated in his description of the Barasana people. Goody quotes the observation of Stephen Hugh-Jones that the Barasana people are ‘totally uninterested in wildflowers’. However, in the very next sentence, he writes that they use wildflowers as body decoration, usually wearing them as earrings or over the ear (p. 20).

As for environmental factors, there are non-agricultural societies living in areas where flowers are scarce, yet they still find aesthetic value in them. For example, the traditional Inuit societies of the arctic part of the Nunavut region of Canada use flowers as decorations (Norton, 2019). Nomadic Bedouin tribes in the deserts of southern Israel use intricate decorative floral motifs in their traditional embroidery (Fouze & Amit, 2019). The same applies to the traditional clothes of the Sámi people of northern Scandinavia (KulturIT, 2020 refers to the online depository of historical photographs and items from Scandinavian museums).

There have been observations of extremely flower-loving hunter–gatherer societies, such as the Lanoh people living in the mountains of Malaysia's upper Perak region. Flowers appeared as their most common decoration and in their origin myths (Evans, 1913, pp. 71, 159, 169). Flowers also play a role in the most important traditional head-hunting rituals of the Puyuma people in Taiwan (Cauquelin, 1991, p. 144). Some Micronesian societies were socially homogenous and without advanced agriculture, yet flowers were a vital part of their culture (Linton, 1926, pp. 64–67). Furthermore, flowers were used during burials in pre-agricultural societies (Nadel et al., 2013) and possibly even among the members of *Homo neanderthalensis* (Solecki, 1975), but see Sommer (1999).

These examples are not exhaustive. They only illustrate that an aesthetic appreciation of flowers appears even in extreme environments and that non-agricultural societies can also develop a complex and rich ‘culture of flowers’. Flower-loving agricultural societies appeared independently in the Middle East, Eastern Asia and Central America. It seems improbable that all three cultures would independently choose flowers as their prominent aesthetic luxury items without any previous tendency towards their appreciation.

To conclude, we think it would be premature to say that the aesthetic appreciation of flowers is a purely cultural phenomenon. It is conceivable that there is an evolved universal tendency to appreciate flowers aesthetically, but that this tendency differs in the extent of its manifestation across cultures. The theory of Orians and Heerwagen tries to shed some light on the origin of this underlying tendency.

### 5.6. General discussion

We can summarise that if there was any influence of shape, colour or edibility on the aesthetic responses to fruits or flowers in our sample, it was probably in favour of fruits in Study A (Czech plants). Still, the fruits and flowers in this study were rated similarly.

In Study B and C (African plants), the raters’ unfamiliarity with the fruits and the higher number of fruits with a less liked green colour might have lowered their rating. However, the flowers were equally unknown to the raters, and they also featured some of the generally least attractive colours (yellow). Moreover, the preference for flowers was so overwhelming that it could not have been caused only by the differences in colours. For example, when we removed the photographs with the least preferred and predominantly fruit colours – green and brownish yellow (seven fruits and one flower) – and then compared the mean ratings for all flowers and fruits again, the difference was still substantial: 0.51 points (0.70 points before the removal). Furthermore, the habitat selection theory assumes that flowers and fruits elicit aesthetic responses because they represent cues of resource availability. From this point of view, once flowers and fruits are distinguished from other types of objects, their colour or shape should not be that important.

Most people would probably describe attractive fruits as tasty or delicious and attractive flowers as beautiful. The aesthetic responses to flowers thus seem to be related to characteristics other than food availability, especially in a direct way. Flowers should serve as predictors of food availability in the future, but the same also applies to fruits. It is questionable whether a group of gatherers should explore and remain in an environment full of flowers that will turn into edible fruits or nuts in a month or even longer. It might be more beneficial to choose a place with ripening fruits that promise a source of nutrition at the given moment and also in subsequent weeks.

One may argue that flowers helped our ancestors to identify not only the presence of food but also other useful resources such as medicine. However, the same also applies to fruits or at least to the conspicuous ones we used as stimuli. Nonetheless, an important source of energy for African hunters-gatherers and African primates is underground tubers. Flowers of these plants might be beneficial in identifying their exact location. The problem is that geophytes usually bloom only once a year and for a short period. Therefore, it would be useful to operate with flowers as location cues only over very long timescales simply because they are not present for most of the year. Another factor that might play a significant role in the aesthetic appreciation of flowers is seasonal change; flowers represent proof that the dry period (or winter) is over. However, positive seasonal changes are not related solely to flowering plants, but also with the change of weather and temperature, the migration and awakening of animals, and especially with the growth of fresh leaves and other green parts of plants. This complex of environmental changes undoubtedly triggers human aesthetic feelings. Nevertheless, we think that a single cue (flowers) is a weaker stimulus than fruits. Fruits represent some of the most important human food sources, and they too serve as indicators of other resources and seasonal changes (although possibly not as strongly as flowers).

Flowers that try to attract pollinators are generally considered conspicuous. A general assumption is that they are probably more conspicuous than fruits. This might be correct, but to our knowledge, there is no work that would compare the conspicuousness of flowering and fruiting plants. Flowers are more numerous than fruits (because not all flowers survive until the formation of fruits), and they sometimes appear before leaves. However, fruits are usually larger than flowers and sometimes remain on plants even after the leaves fall. Many plant species use animals for seed dispersal, so they also need to attract them. Many primates (including humans), as frugivorous organisms, should be adapted to localise fruits effectively. Some authors suggest that primates’ trichromatic vision evolved as an adaptation to distinguish ripening fruits from their background (Osorio & Vorobyev, 1996; Párraga et al., 2002; Regan et al., 2001; Sumner & Mollon, 2000). Moreover, in Study A, raters also evaluated photographs of the whole plants. The difference in the overall conspicuousness of flowering and fruiting plants should be very important in this case, especially when it was quite difficult to distinguish flowers from fruits at such a distance. Still, we observed no differences in the aesthetic response. Therefore, it is not certain that flowers are favoured over fruits because of their conspicuousness.

The fact that we found a stronger aesthetic response (Study B) and preference (Study C) for flowers in the sample from African savannas but not in the Czech sample is worthy of attention. It might indicate that the preference for flowers is more pronounced for plants related to the supposed ancestral environment (although its localisation to African savannas has been widely criticised). Another explanation is that fruits are more context-dependent than flowers during aesthetic evaluation. Redies (2015) nicely illustrated the importance of context. In his model of visual aesthetic experience, he distinguishes stimulus processing in perceptual and cognitive channels. The perceptual channel focuses on the object's physical properties (such as shape or colour), while the cognitive channel operates with contextual information (such as familiarity or prototypicality). Aesthetic experience usually occurs when there is an appropriate response in both channels. Emotional processing can further modulate the extent of aesthetic experience. It seems that flowers have a strong *aesthetics of perception*, whereas fruits rely more on the *aesthetics of cognition*. In other words, flowers might be attractive for their appearance and fruits for what they mean. However, it remains an open question why flowers should be preferred on the perceptual level. It is possible that the information processing of flower stimuli has greater efficacy (maximising information transmission) and efficiency (information processing at low metabolic costs) than that of fruit stimuli. In such a case, the aesthetic response to flowers might be a by-product of processing bias (see Renoult and Mendelson, 2019 for details about this concept of information processing). Therefore, it would be highly beneficial to study possible differences between flowers and fruits from the perspective of information processing.

It is also possible that aesthetic responses towards flowers are not a human adaptation or even a purely cultural phenomenon. On the contrary, it could be an ancestral trait that humans share with other primates. As mentioned above, flowers are nutritionally important for some primate species.

Even though our results do not support the hypothesis we derived from the habitat selection theory, this certainly does not mean that a connection between flowers as cues of resources and the aesthetic responses towards them does not exist. Such a connection most likely plays some role in the aesthetic response to both flowers and fruits. The link to fruits is simple and straightforward because they are considered as food. Our results support this notion because the familiarity with fruits and knowledge about their edibility probably played an important role in their rating. On the other hand, the connection between flowers and resources is more intricate. Our data show that other factors probably exist that further enhanced the attractiveness of flowers throughout human evolution; as we discussed, cultural differences and information processing might be among them.

## 6. Limitations and future directions

We used photographs in this study because it was impossible to display real plants in the flowering and fruiting stage simultaneously. However, the use of photographs is widespread in this type of research, and our previous results showed no substantial differences between the rating of real flowers and that of their photographs. Nevertheless, we might get more in-depth insight by designing an experiment with various real flowering and fruiting plant species in natural settings. We could also abandon the requirement of fruit edibility to be able to prepare a set of stimuli with fully balanced colours and shape properties of flowers and fruits.

It would undoubtedly be beneficial to repeat the study with different stimuli to ensure that the results were not mere artefacts owing to some unknown methodical error.

Cultural aspects certainly modify human attitudes towards flowers. The study should be repeated in other cultures, especially non-agricultural ones, to reveal to what extent our results are culture-dependent. Even among agricultural societies, there might be differences that influence their aesthetic responses to flowers and fruits, such as religion, educational system, traditions, landscape, etc. We cannot generalise our conclusions based on studies performed on a single society. We would like the readers to consider this paper as a mere first step that should draw their attention to this issue and encourage further research.

Future research should also focus on other factors that might account for human attraction towards flowers, especially efficacy and efficiency in information processing. We should explore whether such attraction is only a by-product of generally preferred shapes and colours or whether there are some characteristics (or their combinations) unique to flowers that make them beautiful to human eyes. We should also explore the role of cognitive processing (context) in the aesthetic evaluation of flowers and fruits, for example, by using a mixture of known and unknown or edible and poisonous stimuli.

## 7. Conclusion

The habitat selection theory of Heerwagen and Orians offers an attractive yet never empirically tested explanation for the origin of human aesthetic appreciation of flowers: the relation of flowers to food and resource availability. Our data imply that human aesthetic responses to flowers cannot be explained solely by this factor, although it might play some role. However, we urgently need data from a broader cultural sample to confirm our limited findings. The question of why humans tend to aesthetically appreciate flowers seems to remain partly unexplored. The habitat selection theory proposes a basis for further investigations of other factors, such as information processing, that could extend this theoretical framework. The issue is crucial for anyone who studies people–plant interactions and deserves renewed attention from researchers working in related fields.

## Data Availability

The data associated with this research are available at https://doi.org/10.6084/m9.figshare.11956632.v1

## Research transparency and reproducibility

Analyses reported in this article can be reproduced using the data provided by Martin Hůla (2020): https://doi.org/10.6084/m9.figshare.11956632.v1
